# The experience of motherhood in a container city: the effects of the earthquake and changing needs

**DOI:** 10.3389/fpsyg.2026.1924330

**Published:** 2026-09-25

**Authors:** Feyza Ucar Cabuk, Seda Eskidemir, Kübra Engin Bayram, Merve Karakaş Aksoy, İlkay Ulutaş, Fatma Tezel Şahin

**Affiliations:** 1 Department of Preschool Education, Mersin University, Mersin, Türkiye; 2 Program of Child Development, Akdeniz University, Antalya, Türkiye; 3 Center for Career Development, Application, and Research, Balikesir University, Balikesir, Türkiye; 4 Department of Child Development, Faculty of Health Sciences, Atilim University, Ankara, Türkiye; 5 Department of Preschool Education, Gazi University, Ankara, Türkiye

**Keywords:** child, earthquake, mother, needs, parenting

## Abstract

**Introduction:**

The twin earthquakes that struck on February 6, 2023, centered in Kahramanmaraş/Türkiye, caused widespread destruction in the region and completely transformed daily life. This study aims to examine the earthquake’s impact on children, the experience of motherhood following the earthquake, the effects of life in a container city on this process, and the needs of mothers and children.

**Methods:**

Data were collected through semi-structured interviews with 37 mothers living in a container city in the region who have children aged 4–6.

**Results:**

Data analysis revealed that after the earthquake, children developed emotional, behavioral, and physical reactions, while mothers faced parenting challenges related to behavior management, overprotection, and quality time. Additionally, it was observed that the conditions in the container city were unsafe, leading to restrictions on the children; the cramped nature of the container area and the lack of personal space strained family relationships; and the chaos and responsibilities placed significant pressure on the mothers.

**Conclusion:**

Mothers expressed that they personally need emotional and social support, as well as training on emotional and self-regulation regarding their children, safe environments, positive social relationships, self-care, self-confidence, and communication with their children. They also expressed a need for training on parental attitudes and behaviors, child psychology, health, and development, addictions, attachment, safety, privacy, special needs, and structured activities.

## Introduction

Earthquakes are considered a traumatic disaster in terms of loss and severity (Dai et al., 2016). In addition to their physical destructiveness, earthquakes can profoundly affect emotional and mental health because they completely disrupt an individual’s daily functioning. These effects can vary depending on the intensity, duration, and magnitude of the earthquake’s destruction, as well as the efforts made to normalize the situation afterward. It is stated that the living conditions of survivors, the logistical and psychological support provided to them, security, and the sustainability of this support play a significant role in reducing trauma or post-disaster stress (Selzler and Grandbois, 2011).

In addition to physical damage, earthquakes can also increase the likelihood of mental health issues such as depression and post-traumatic stress disorder (PTSD) (Dai et al., 2016; Iqbal and Sheikh, 2023). Studies conducted after the earthquake have shown that high rates of post-traumatic stress disorder, anxiety, and behavioral problems emerge among children affected by the earthquake (Atabey et al., 2025; Eroğlu and Yaksı, 2025; Kolaitis et al., 2003; Kurt et al., 2024). Karaoğlu (2025) observed that children who experienced the earthquake reflected their fears, losses, and desire to return to their normal routines in their drawings. Kolaitis et al. (2003) found that parents continued to experience anxiety and depression 1 year after the earthquake, and this situation influenced the emergence of psychosocial problems in children (Kerimoğlu Yıldız et al., 2024).

Due to its location in an earthquake zone, Türkiye frequently faces earthquake-related disasters. On February 6, 2023, two devastating earthquakes measuring 7.7 and 7.6 magnitude, centered in Kahramanmaraş, directly affected 11 provinces and resulted in the loss of 50,783 lives. The twin earthquakes have affected 13.5 million Turkish citizens, including 4.5 million children, and 1.7 million registered refugees, including 811,000 children. The massive scale of the loss and the need for support affected the entire country (AFAD, 2023). Shelter areas provided temporary shelter, such as tents and containers, along with logistical and psychological support. Following the earthquake, it was revealed that over 675,000 people were housed in 392 official container sites across 11 provinces. While earthquake survivors are being relocated to permanent housing, many temporary settlements remain in active use (AFAD, 2023).

Although psychosocial support was provided in temporary settlements immediately after the earthquake, the earthquake’s magnitude prevented the content and organization of these services from being established and maintained. It is reported that approximately 4 million children - including 390,000 migrant children-face restricted access to education, and that children’s nutritional needs are steadily increasing (UNICEF, 2024).

Recently, earthquake survivors have been gradually moving into permanent housing, and psychosocial support services may be disrupted due to both the dispersal of survivors across different settlements and limited financial resources. In fact, earthquakes destroy the ecosystem in which individuals live and force them to enter a new ecosystem (Bronfenbrenner, 1979; Fiala et al., 2025). While the traumatic break from previous systems allows some families to spontaneously accept the new system, in others, attachment to the past and the differences between the new and old environments may reduce this acceptance and adaptation. In this context, mothers bear a greater psychological burden in the role of both maintaining their own existence and supporting their children’s existence (Thurston et al., 2021; Ulutaşdemir and Kabakuş Aykut, 2023).

It is noted that the psychological impact of the February 6, 2023, earthquakes on children and families persists, contributing to an increased risk of violence, abuse, and exploitation (UNICEF, 2024).

While post-earthquake reports provide important information on living conditions in container cities, they also highlight the need to support earthquake survivors in feeling safe and understood and to address their anxieties about the future. Children use a range of coping strategies to manage distress and restore well-being during and after disasters, including escape, social isolation, submission, resistance, problem-solving, and seeking social support (Raccanello et al., 2024). Building on this literature, Powell et al. (2026) identified three broad categories of children’s disaster coping strategies: positive strategies, negative strategies, and strategies that may be adaptive or maladaptive depending on the context. Positive strategies include positive reframing, seeking support, play, community and peer connection, engagement, acceptance, self-efficacy, emotion regulation, competence, coping, and self-reliance. Negative strategies include denial, avoidance, social isolation and withdrawal, rumination, emotional suppression, blaming others, substance use, self-blame, fatalism, and magical thinking. Some strategies, such as distraction, religious coping, and problem-solving, may function adaptively or maladaptively depending on the context in which they are used (Powell et al., 2026). Consistent with this distinction, Raccanello et al. (2024) reported that the use of negative coping strategies is associated with more negative psychological symptoms, whereas positive coping strategies are associated with more adaptive outcomes. Children’s ability to adopt and maintain adaptive coping strategies may, in turn, be influenced by the support they receive from their parents. Parents play a central role in children’s coping and adjustment during and after disasters by providing emotional security, helping children interpret and make sense of stressful experiences, and modeling appropriate responses to adversity (Masten and Osofsky, 2010).

### Theoretical framework

Earthquake trauma does not operate at a single level; it simultaneously disrupts the environments children depend on, the relationships that regulate them, and the family systems that sustain them (Masten and Osofsky, 2010). Bronfenbrenner’s Ecological Systems Theory, attachment theory, and Bowen’s Family Systems Theory provide interconnected lenses for understanding how these disruptions unfold and compound one another in the post-disaster context (Bowen, 1978; Bowlby, 1969; Bronfenbrenner, 1979).

Bronfenbrenner’s Ecological Systems Theory conceptualizes child development within five interconnected systems: the microsystem, encompassing the child’s immediate environment and relationships; the mesosystem, representing the interactions between these environments; the exosystem, covering external contexts that indirectly affect children and families; the macrosystem, reflecting broader cultural, social, economic, and institutional influences; and the chronosystem, capturing the role of time, life transitions, and historical events in development (Bronfenbrenner, 1979; Bronfenbrenner and Morris, 2006). According to this theory, the effects of natural disasters extend beyond the individual and influence the broader ecological systems surrounding children and families (Bronfenbrenner, 1979). Following an earthquake, the microsystem–the child’s immediate environment, including the home, neighborhood, school, and daily relational settings–may be disrupted or destroyed. This disruption represents more than a physical or logistical loss, as the microsystem constitutes the primary context in which children develop feelings of safety, stability, routine, and belonging. Moreover, the family system’s response to disaster is shaped not only by changes within the microsystem but also by the interconnected influence of broader ecological layers, including the mesosystem, exosystem, macrosystem, and chronosystem (Fiala et al., 2025). In the context of the Kahramanmaraş earthquakes, with magnitudes of 7.7 and 7.6, the exosystem, macrosystem, and chronosystem provide particularly important guidance. The exosystem helps reveal how earthquake-related disruptions in parents’ employment, income, and access to services affect children’s development and family functioning. The macrosystem enables the examination of broader social, cultural, economic, and institutional conditions that shape families’ experiences and recovery after a disaster. The chronosystem frames the earthquake as a significant historical transition, whose effects may unfold over extended periods and interact with subsequent developmental and family changes (Bronfenbrenner, 1979; Bronfenbrenner and Morris, 2006; Fiala et al., 2025).

The mother–child attachment relationship is where the effects of ecological disruption may become most visible (Ainsworth, 1969; Bowlby, 1960). Attachment theory suggests that children are biologically predisposed to seek proximity to caregivers in situations involving fear, uncertainty, or threat. According to John Bowlby, children are born with instinctive attachment behaviors directed toward a primary caregiver, serving a critical function for survival and emotional security (Bowlby, 1969). Following disasters such as earthquakes, children may lose not only their physical environments but also the relational and environmental stability that supports emotional security. When children’s microsystems are disrupted, attachment figures–most often mothers–become central sources of safety and regulation. Under such conditions, children may display increased attachment-seeking behaviors, separation anxiety, emotional dysregulation, and heightened dependency as they attempt to cope with uncertainty and insecurity (Bowlby, 1960; Siegel, 2012).

During and after a disaster such as an earthquake, family members tend to turn to one another because safety can also be understood as a means of establishing connections with others, particularly through close relationships (Williamson et al., 2021). Additionally, a family member’s attachment style shapes how they interact and behave after a crisis such as an earthquake. However, human attachment is dynamic (Siegel, 2012). This dyadic pressure does not remain contained within the mother–child relationship but can spread through the entire family system. According to Bowen’s Family Systems Theory, the child and caregiver are inextricably linked and embedded within a larger system (Cox and Paley, 1997). All subsystems within the family form their own systems yet remain interdependent through mutual relationships and interactions (Bowen, 1978). During crises such as earthquakes, family members’ responses vary depending on their level of emotional differentiation, communication patterns, and the system’s flexibility. Following disasters, marital tension, sibling conflict, and difficulties in family communication and emotional regulation may increase. In this process, the distress experienced by one family member may affect the well-being of others (Bowen, 1978; Fiala et al., 2025).

### The effects of earthquakes on children and families

The disruption caused by earthquakes can continue beyond the immediate event as families attempt to adapt to new living conditions. Survivors must not only stay alive but also find roles within the new context created for them. In container cities, the temporary nature of this environment may delay the development of belonging and trust and may also slow post-traumatic recovery (Fiala et al., 2025; Prime et al., 2020; Yumbul et al., 2018).

Families remain separated from stable homes, familiar neighborhoods, and regular school environments, while overcrowded and insecure living conditions may restrict privacy and emotionally supportive interactions. As a result, families may experience not only acute post-earthquake trauma but also prolonged and chronic stress associated with temporary shelter conditions (Kaya et al., 2026; Kerimoğlu Yıldız et al., 2024). These conditions may also affect family relationships and parenting. Following disasters, marital tension, sibling conflict, and difficulties in family communication and emotional regulation may increase. One family member’s distress may affect others’ well-being (Bowen, 1978; Fiala et al., 2025). Parents experiencing post-traumatic stress may have difficulty spending time with children and maintaining caregiving practices (Kaya et al., 2026). Feelings of helplessness, emotional withdrawal, avoidance, rejection, or excessive fear of losing children may negatively affect parenting behaviors and emotional responsiveness. Such difficulties may, in turn, intensify children’s emotional and behavioral reactions following trauma (Kerimoğlu Yıldız et al., 2024). Motherhood is not solely a caregiving role but also a socially constructed position shaped by gendered expectations and cultural norms (Kağıtçıbaşı, 2007). In Türkiye, mothers have traditionally taken greater responsibility for raising and caring for children than fathers (Erkal et al., 2007). Traditional gender roles have contributed to the belief that housework and childcare are mainly women’s responsibilities. Although social, economic, and cultural changes have led to more egalitarian views about gender roles, traditional expectations still influence women’s experiences of motherhood and family life. Similarly, Kaymaz et al. (2026) found that while women increasingly hold more egalitarian views about motherhood and family roles, traditional gender expectations continue to shape family life. Consequently, mothers often remain the primary caregivers and an important source of support for children, particularly during stressful and challenging experiences such as disasters. This caregiving role may become particularly demanding following an earthquake. Women in disaster settings frequently experience increased caregiving burdens, reduced social support, psychosocial difficulties, and limited access to institutional resources (Ulutaşdemir and Kabakuş Aykut, 2023). Within attachment relationships, mothers often function as children’s primary source of emotional regulation and security, particularly during periods of uncertainty and threat (Thurston et al., 2021). Following disasters, these responsibilities may intensify as environmental supports are disrupted and mothers become children’s most immediate source of stability. At the same time, mothers may be attempting to manage their own trauma, grief, uncertainty, and post-disaster stress while continuing to provide care and emotional support. Therefore, mothers’ psychological well-being plays a critical role in children’s post-disaster adjustment and recovery (Bozkurt Karalı and Özkan, 2025; Kerimoğlu Yıldız et al., 2024). Supporting mothers is closely connected to supporting children, particularly in the aftermath of a disaster. Mothers’ accounts of earthquake trauma provide important insight into the effects of earthquakes and temporary settlement life on children, family relationships, parenting roles, and support needs. Existing literature has examined children’s mental health and trauma responses following earthquakes (Cénat et al., 2020; Eroğlu and Yaksı, 2025; Khan et al., 2023; Li et al., 2024; Nagae and Nagano, 2023; Pfefferbaum et al., 2014). However, understanding mothers’ experiences can provide a broader perspective on post-earthquake family adjustment by highlighting how mothers perceive changes in their children, parenting roles, family relationships, and support needs.

Trauma-informed approaches highlight the importance of understanding caregivers’ experiences and perceived needs when developing support programs (Yumbul et al., 2018).

However, several gaps remain in the literature. First, temporary shelter settings are often treated as background conditions rather than as environments that actively shape parenting and family life. Second, early childhood, particularly ages 4–6, remains underrepresented in post-disaster family research despite its developmental importance. Third, existing studies have focused mainly on child outcomes or parental distress, with limited attention to mothers’ lived parenting experiences and perceived support needs within temporary settlements. To address these gaps, the present study examines the parenting experiences of mothers living in container cities following the February 6 earthquake, with particular attention to early childhood and support needs. Findings based on mothers’ own narratives are expected to contribute to both parenting and disaster literature and to guide the development of family- and early childhood-focused intervention programs. Accordingly, the study seeks to answer the following research questions:

1. How do mothers living in a container city perceive and experience the effects of the post-earthquake period on their parenting practices?
2. How do mothers living in a container city perceive and describe changes in their children’s behaviors following the earthquake?
3. What support needs do mothers living in a container city perceive for themselves and their children during the post-earthquake period?

## Materials and methods

### Research design

This study used a qualitative descriptive research design and employed hybrid inductive-deductive thematic analysis (Braun and Clarke, 2006; Fereday and Muir-Cochrane, 2006). Descriptive thematic analysis is a qualitative research method that reveals patterns (themes) in data derived from participants’ experiences (Sandelowski, 2000). This approach was deemed appropriate for examining mothers’ perceptions of their motherhood experiences and post-earthquake needs. Through thematic analysis, the aim was to identify and interpret recurring meanings and patterns in the participants’ narratives (Braun and Clarke, 2021).

Descriptive thematic analysis was conducted in a hybrid manner using deductive and inductive coding processes (Thomas, 2006). Deductive coding aimed to determine perceptions of the disaster’s impact on maternal and child needs through research questions shaped in line with the literature; Inductive coding, on the other hand, allowed additional themes to emerge directly from the participants’ narratives (Patton, 2015).

### Participants

The study group comprises 37 mothers with children aged 4–6 who were affected by the February 6, 2023, earthquake and still live in the temporary living area created after the earthquake. Criterion sampling method was used to determine the study group. This method enabled the identification of criteria aligned with the research objective (Patton, 2015). Accordingly, the inclusion criteria for the study were as follows: participants were required to have experienced the February 6 earthquakes, reside in a container city in Osmaniye/Türkiye have at least one child aged 4–6 years, and be able to communicate in Turkish.

Sample size in qualitative research varies according to the purpose of the study, the utility and credibility of the data, and the feasibility of data collection within existing time and resource constraints Sample size varies depending on the purpose of the research, what is useful and reliable, and what can be accomplished within available time and resources (Marshall and Rossman, 2006; Miles et al., 2013; Patton, 2015). In this study, sample size was evaluated based on data saturation and thematic significance level (Braun and Clarke, 2021; Merriam, 2018). During the interviews with 37 mothers, no substantially new codes or themes emerged after the 30th participant, indicating that thematic saturation had largely been achieved. However, data collection continued until all planned interviews were completed to increase the depth, variation, and confirmability of the findings by including diverse participant experiences (Creswell and Poth, 2018; Merriam and Tisdell, 2016).

The mean age of the 37 mothers was 33.07; 21 had completed primary school, 15 had graduated from high school, and 1 held a university degree. Participants have worked in low-income occupations and relied partially on government financial assistance. Regarding the number of children of the participating mothers, 10 had 1 child, 13 had 2 children, 9 had 3 children, and 5 had 4 children. Additionally, 3 mothers lost a relative in the earthquake. The mothers have been living in the container city for approximately 22 months.

### Data collection tools

Data collection was carried out through semi-structured face-to-face interviews. The researcher developed the questions by drawing on previous research and aligning them with the study’s objectives (Fu et al., 2020; Jennings et al., 2018; Keleş, 2024; Kimura, 2020; Kobayashi et al., 2020; Yoosefi Lebni et al., 2020; Yumbul et al., 2018). The literature review revealed that mothers’ needs are concentrated in the areas of care, attitude, health, safety, and education; therefore, questions were formulated to identify these needs. Field experts (from early childhood education, child development, assessment and evaluation, and educational programs) reviewed the questions for suitability, scope, and comprehensibility.

In addition, the researchers conducted a pilot study with six mothers to evaluate the applicability of the interview questions and the interview process. The pilot study revealed that some questions generated similar responses, certain questions were difficult for mothers to answer due to language barriers, and the overall interview duration was lengthy. Accordingly, we reduced the number of interview questions. In addition, we revised and clarified some questions to support comprehension and expression during the interviews. For instance, because several mothers had difficulty responding to the question, “What kind of parent do you think you are?,” we added sample statements to support understanding. Following the pilot study, the interview form was finalized with 11 questions. In conclusion, the questions included in the interviews were as follows: How does a typical day with your child at the temporary shelter go? What changes did you observe in your child’s behavior after the earthquake? What changes did you observe in your attitudes and behaviors toward your child after the earthquake? What can you say when evaluating this living area in terms of your children?

### Data collection

Data collection was conducted between October 15, 2024, and January 15, 2025, following ethical approval and the completion of all required official permissions. As the study was conducted within the scope of a larger project, the project approval process and the time required to obtain the necessary institutional permissions influenced the timing of data collection.

Because the participants lived in a container city in Osmaniye province, we first contacted the container city management. We explained the study’s purpose and progress and obtained official permission to reach the participants. Through announcements, posters, and visits to the containers, we invited mothers with children aged 4–6 to participate. We organized a meeting with mothers to explain the study’s purpose. Mothers who agreed to take part in the study provided informed consent forms.

Data collection was carried out through individual interviews with the mothers. The mothers’ preferences were considered when determining the timing and location for the interviews. Some mothers preferred interviews to be conducted in their own containers, while others found meeting rooms or the youth center in the common area more suitable. Some mothers preferred common areas (meeting rooms, activity areas) because they felt more comfortable there and wanted to avoid disturbing their household members.

Interviews were conducted using an interview protocol designed to support the validity and reliability of the data collection process. Establishing trust-based communication between the researcher and participants enabled mothers to express themselves more comfortably and share their experiences in greater detail, thereby supporting the credibility of the study (Merriam and Tisdell, 2016; Patton, 2018). The protocol included the following stages: welcome, introduction to the purpose of the research, ethical information, questions regarding experiences, and closing (Creswell and Poth, 2018; Patton, 2018). The stages of the interview protocol are presented in Table 1.

**TABLE 1:** Protocol stages of the interview.

Stage	Application
Welcome	Participating mothers were greeted warmly and with a friendly expression in a quiet and calm environment where participants could feel comfortable, after necessary preparations were made.
Information about the purpose of the research	Participants were informed that the aim was to determine their needs after the earthquake, and mothers were given information about the study.
Ethical information	Participants were informed that the research was conducted in accordance with ethical rules. Informed consent was obtained from participants regarding their voluntary participation in the research and the recording of their voices; they were informed that they could stop the recording or end the interview at any time. It was also stated that participant identities would be kept anonymous and that the data obtained would be protected in accordance with the principle of confidentiality.
Questions	Semi-structured questions were asked to the participants, and their experiences were recorded.
Closing	The participating mothers were asked if they had any additional opinions or statements they wished to add. They were thanked for their valuable contributions to the research, and the interview was concluded.

Accordingly, as specified in the protocol, the interviews were conducted in settings such as meeting rooms or youth centers and scheduled when no other activities were taking place. The interview environment was arranged to provide a quiet atmosphere and to allow the researcher and participant to sit facing one another, thereby ensuring an uninterrupted interview process.

To prevent mothers from reliving past traumas and triggering their traumas, the literature on qualitative studies conducted with traumatized participants was reviewed (Alessi and Kahn, 2023; Karmakar and Duggal, 2024). Accordingly, care was taken to ensure the mothers felt safe during the preparation of interview questions and the conduct of the interviews. Mothers were informed that they could end the interview, take breaks, and remove any statements they did not wish to be recorded. A non-judgmental, supportive, and open-ended communication style was used throughout the interview process, with encouraging statements included to help participants express themselves. The questions focused not on the details of the earthquake itself, but rather on the support the mothers provided to their children after the earthquake, their current needs, and their coping processes. Therefore, instead of recalling past traumatic experiences in detail, the questions were structured to address the participants’ current situations and future needs.

An interview lasted an average of 20–30 min. Researchers avoid judging participants verbally, through gestures, or by facial expressions during the interview.

### Data analysis

The data analysis was conducted using a hybrid approach of deductive and inductive coding in line with the descriptive thematic analysis design of the study (Braun and Clarke, 2006). This approach was preferred because the study aimed to analyze participant experiences within the scope of the research question while also allowing new meanings, themes, and experiences to emerge directly from the participants’ narratives. The analysis proceeded in six stages following Braun and Clarke’s (2006) framework.

In the first stage, the research team read all interview transcripts. Recurring expressions, emotionally significant moments, and content relevant to the research questions were marked.

In the second stage, initial codes were generated across the dataset. Some codes were developed deductively, guided by the research questions and the theoretical framework (Proudfoot, 2023). For example, codes related to the observed effects of earthquakes on children’s behavior, mothers’ parenting experiences, time spent with children, and mothers’ educational expectations were derived from interview questions and the existing literature. Other codes emerged inductively from the data itself (Braun and Clarke, 2006). For example, fear of losing the child and increased overprotection, inability to feel at home in the container city, loss of patience and tolerance in parenting, differences in how younger and older children were affected, and safety concerns specific to the container city setting were not anticipated in advance but arose from participants’ own accounts. Throughout this process, each statement was examined at the level of meaning units. Where the same data segment was relevant to more than one code, overlapping coding was permitted.

In the third stage, codes were grouped under potential themes. The relationships between codes were mapped, and decisions were made about which codes were strong enough to form an independent theme and which should remain as subcategories. For example, codes such as irritability, anger, fear, and sleep disturbance were initially identified separately but were later merged into a single theme addressing the emotional and behavioral effects of the earthquake on children. Similarly, codes related to space constraints, safety concerns, lack of privacy, and inability to establish routines were grouped under the theme of container city conditions.

In the fourth stage, each theme was checked against the coded data extracts and then against the full dataset. A thematic map was developed. Some themes were restructured, and codes that lacked sufficient data support were placed within related themes rather than treated as independent themes. The boundaries between themes were clarified, and each theme’s relationship to the research questions was reviewed.

In the fifth stage, themes were defined and named. Four main themes were identified: the effects of the earthquake on children, parenting experiences following the earthquake, the effects of container city living on parenting and child behavior, and mothers’ support and educational needs.

In the sixth stage, the themes were interpreted in relation to the research questions and the theoretical framework. The findings were connected to ecological systems theory, attachment theory, and family systems theory. Illustrative data extracts were selected to represent each theme and were presented in the findings section. Care was taken to reflect the data’s diversity and to include each participant’s voice in the analysis. Examples of life in the container city were shown in Figure 1.

**FIGURE 1:**
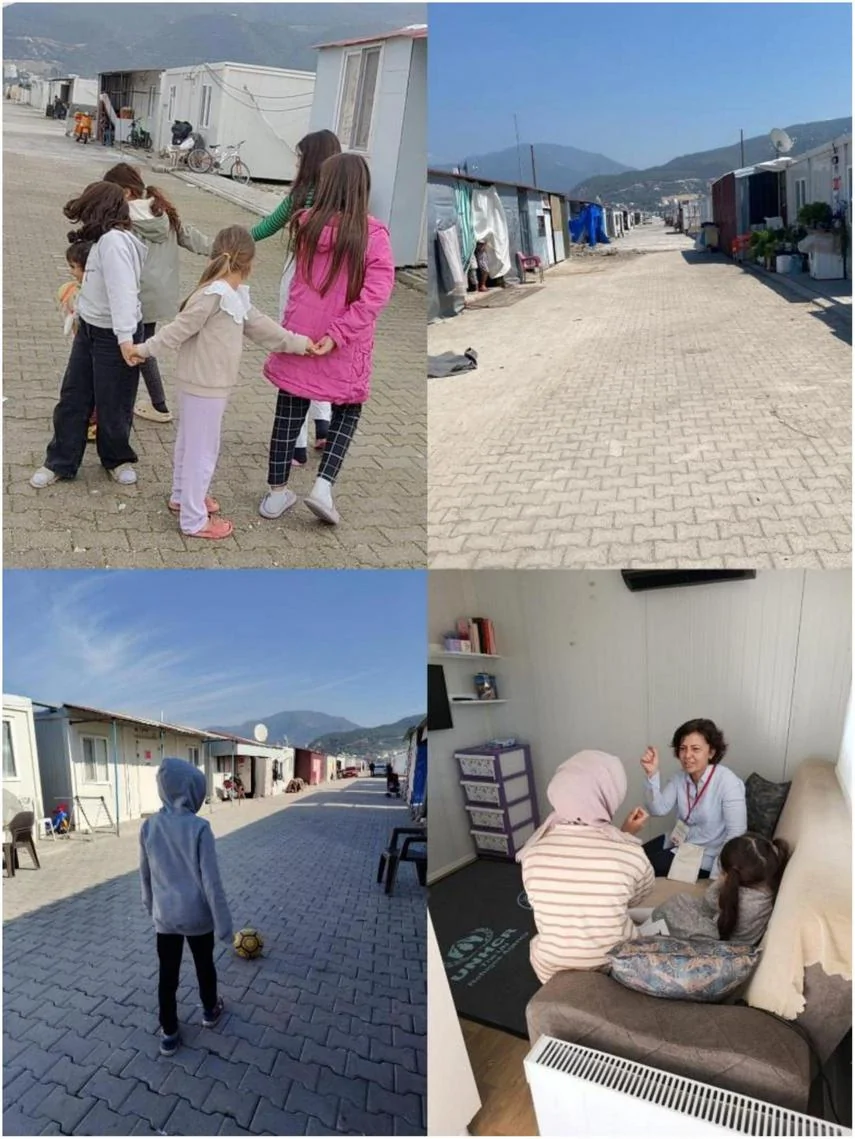
Examples of life in the container city.

### Validity and reliability

In qualitative interviews, the researcher’s neutrality is crucial for data reliability, as it influences the data collection process (Pezalla et al., 2012; Zhang and Okazawa, 2023). Therefore, researchers maintained a neutral stance during the interviews, providing no guidance.

Various measures were taken during data collection, analysis, and reporting to ensure the research’s validity and reliability. Interview protocols were followed to ensure the interviews were conducted in a qualitative and consistent manner. To evaluate the accuracy and reliability of the data in this study, Lincoln and Guba’s (1985) criteria of reliability, consistency, verifiability, and transferability were used. To increase reliability, interview questions were developed in line with field experts’ opinions, and the data collection process was conducted in accordance with ethical principles. To increase consistency, records of all documents used in the data collection and analysis processes were kept, and documents related to the analysis process were stored in a way that allows access when needed. Researchers performed the coding accurately, free from personal biases. Direct quotations from participants’ statements were included to help readers relate the findings to similar contexts.

### The role of the researcher

Since the role of the researcher is considered crucial in qualitative research, the self-reflection process has been adopted as a fundamental principle in this study. Self-reflection requires researchers to question their own positions, values, and potential biases, and to recognize their impact on the research process. Accordingly, transparency and self-awareness were prioritized in the data collection, transcription, coding, thematic analysis, and reporting processes (Collins et al., 2007). The research team adopted a self-reflective approach by continuously evaluating their own impact throughout the entire process.

The researchers conduct studies on mother-child relationships and the socio-emotional development of children; they also carry out projects for disadvantaged children and provide training seminars for mothers. They also conducted volunteer work with earthquake-affected children and families after the Kahramanmaraş earthquake. Three researchers conducted interviews, and after each one, the team jointly evaluated the process.

Researcher 1 experienced the earthquake in Osmaniye, one of the provinces severely affected by the Kahramanmaraş earthquakes. The researcher’s home sustained substantial damage but did not collapse, and the researcher was not trapped under the rubble or physically injured. The researcher also did not lose a first-degree relative in the earthquake. Nevertheless, directly experiencing the earthquake and witnessing its aftermath gave the researcher firsthand insight into the challenges affected families and children faced. These experiences heightened the researcher’s awareness of the difficulties families faced in the post-earthquake period and contributed to the researcher’s interest in understanding their experiences and support needs. This positionality played an important role in motivating the researcher to undertake the present study. At the same time, the researcher remained aware that personal experiences could shape interpretations of participants’ accounts and therefore approached data collection and analysis with reflexivity, seeking to distinguish the researcher’s own experiences from those expressed by participants.

The researchers’ responsibilities are listed as follows:

All researchers work at different universities and have no relationship with the participants. The first author, with their experience in early childhood education, was responsible for structuring the article, preparing interview questions, collecting data, transcribing audio recordings, and editing the article. The second author prepared the interview protocol, collected data, and interpreted the discussion. The third author analyzed the data and interpreted the findings. The fourth author transcribed audio recordings, created codes, interpreted the findings, and wrote the introduction and discussion sections. The fifth author, with experience in early childhood education, structured the article, managed the interview process, and analyzed and interpreted the data. The sixth author prepared the interview questions.

## Findings

The qualitative data obtained from mothers residing in a container city following the earthquake are systematically organized around three guiding research questions. Thematic analysis yielded four overarching themes aligned with these questions: the impact of the earthquake on children, post-earthquake parenting experiences, life in the container city, and emerging needs. Figure 2 presents the findings.

**FIGURE 2:**
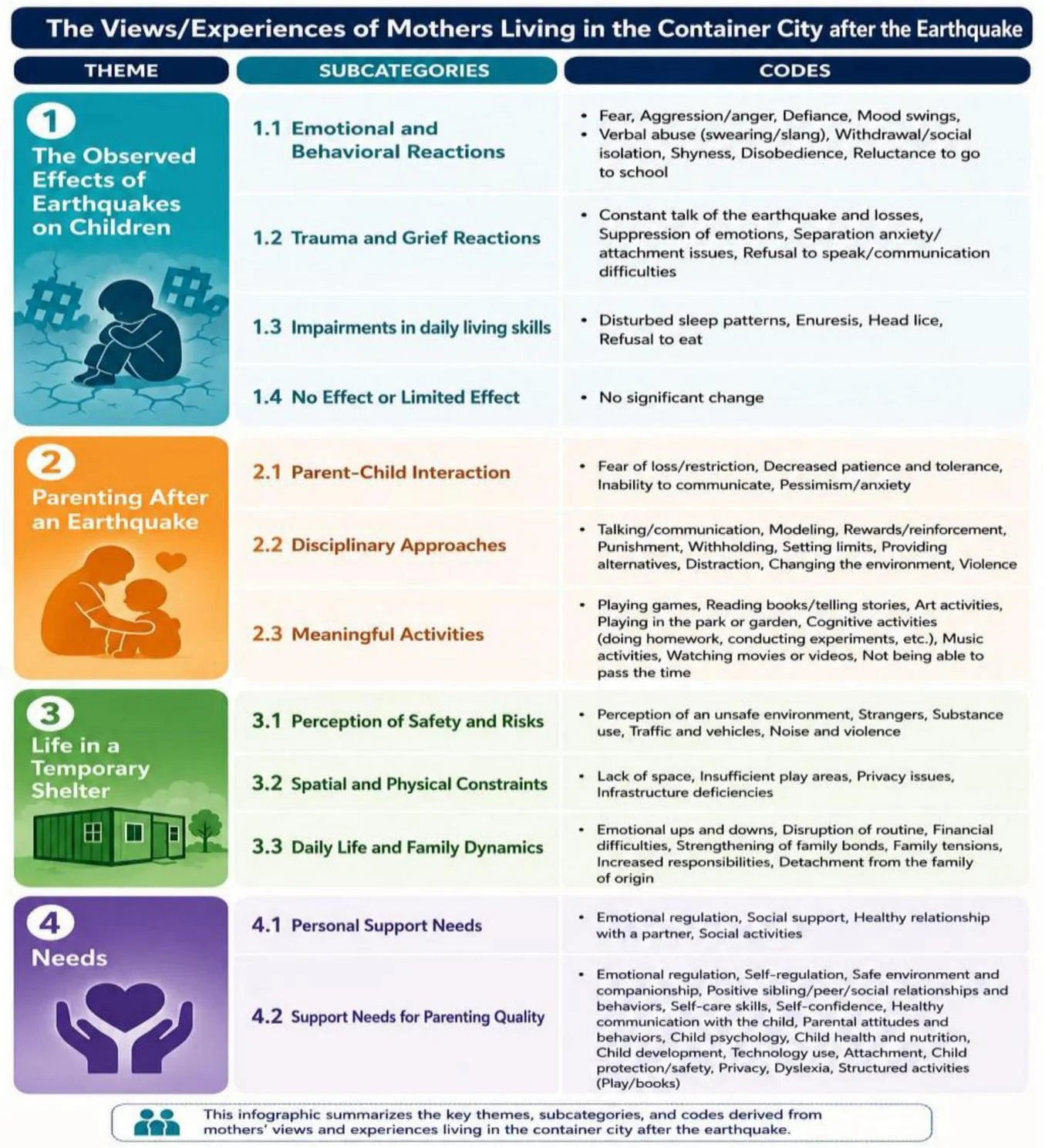
The views/experiences of mothers living in the container city after the earthquake. Artificial intelligence Gemini was used to create the image in this figure.

### Theme 1: the observed effects of the earthquake on children

Interviews with mothers indicate that the earthquake has profoundly affected children. Mothers described these effects as manifesting in emotional and behavioral reactions, trauma and grief responses, and impairments in daily living skills. Many mothers have reported that their children’s behavior has changed since the earthquake, making daily life more difficult. Some mothers have noted sudden and intense emotional shifts, outbursts of anger, and aggressive behavior in their children. These mothers emphasized that their children have become more irritable toward both themselves and their siblings and have started using hurtful language:

*“She’s become very irritable and won’t listen to me. She demands everything while crying. She wasn’t like this at home. She used to be a calm, understanding girl who listened to me. But now she never listens to me. She even swears. That’s why I’m very upset. She’s becoming aggressive, hitting her sibling, and fighting with her sister. I don’t know what to do anymore…” (M34)*

Similarly, some mothers have noted that their children’s fear-based reactions following the earthquake have significantly disrupted their daily lives. Mothers observed that their children did not want to be alone, struggled to be separated from their parents, and associated certain events with the earthquake:

*“For example, when he’s anxious, my son used to be able to go to the bathroom easily before. But now, you know the bathroom in the container–it’s right next to us, inside. But he’s even afraid to go there. ‘I’m scared,’ he says, ‘wait at the door,’ he says. How can I explain it? He doesn’t want to be apart from us.” (M9)*

*“…the container sways when we walk. It’s not like a normal house; he says everything will collapse. He says there’s going to be an earthquake. I think maybe he’s trying to suppress his fear because of the earthquake. After the earthquake, he never sleeps alone. He always wants to sleep with us. He doesn’t want anyone else next to me. I wouldn’t call it jealousy, but I can’t get him to sleep alone; he wants to sleep with me.” (M15)*

Some mothers’ accounts suggest that children struggle to make sense of the losses and trauma they have experienced. Children who have lost a loved one often speak constantly about the person they have lost, harbor the expectation that they will return, and this indicates that the grieving process has not yet been completed:

*“Mom… did she come? … where did she go? Did she become an angel? Did she fly away? She wants to watch her videos and look at her pictures. She misses her so much… He absolutely won’t accept it. It’s as if she’s still here. ‘She was my friend,’ she says. ‘She’s coming, Mom,’ she says. ‘When is she coming?’ he asks. She appears in his dreams. She’d wake up crying all the time. She’d wake up screaming. She says, ‘I saw my aunt, I saw my uncle.’ It was hard for him because we were trapped under the rubble; she was very scared.” (M14)*

Mothers have noted that traumatic experiences are reflected in their children’s drawings:

*“Especially when drawing, they draw houses more often. They ask questions like, ‘Will we have a home? Will we see our deceased loved ones again? Will we be able to see them if another earthquake happens?”’ (M7)*

The earthquake’s effects on children are evident not only behaviorally but also physiologically. Some mothers have stated that their children have lost skills they had previously acquired, particularly noting disruptions in toilet habits, sleep patterns, and eating behaviors:

*“After experiencing the night of the earthquake, my children started wetting themselves. Especially my son, who just started first grade–we still haven’t managed to get past it. He gained toilet control at an early age, but now he sometimes wets the bed as soon as he falls asleep, sometimes in the early morning, sometimes twice… He used to go outside; now he doesn’t want to go inside. They’ve become very irritable; outbursts have started. They didn’t use to respond or use vulgar language. These issues began during the time we’ve been here.” (M13)*

In some children, however, difficulties with speech and self-expression have come to the forefront. Mothers have noted that, despite their children having no intellectual disabilities, they choose not to speak, and this situation is combined with anger and irritability:

*“He’s become fearful; he’s scared of the slightest thing. I was sitting on the second floor, and I think he went into shock because I panicked and rushed him outside during the earthquake. He’s become very attached to me. He won’t leave my side. He’s crying, he won’t speak… He’s irritable, aggressive, and can’t focus on anything. For example, even when the teacher is explaining something, his mind is elsewhere. He’s become fearful. There’s a report stating he can’t speak. There’s no issue with his language, education, or intelligence. He just doesn’t speak. He becomes irritable because he can’t express himself.” (M10)*

On the other hand, some mothers have stated that their children were only minimally affected by the earthquake or that they observed no noticeable changes. These mothers attributed the absence of observable change to their children’s young age. However, attachment theory suggests that even infants are affected by caregivers’ dysregulated emotion and physiology (Bowlby, 1969; Siegel, 2012). The absence of visible behavioral change in very young children should therefore not be interpreted as a lack of impact but may instead reflect the developmental limitations of pre-verbal children in expressing distress.

“When the earthquake happened, my child wasn’t old enough to understand it–she was just a child. I didn’t notice any changes in my child related to the earthquake.” (M33)

*“Nothing much happened to my little girl. Because she was sleeping. There was just a little fear during the earthquake, because she didn’t know what was happening. She was sleeping anyway at that moment. There was a little fear. Yes, other than that, she didn’t feel anything.” (M22)*

Based on mothers’ accounts, it appears that children exhibit emotional, behavioral, and physical differences in their responses to trauma and changing circumstances. These responses align with attachment theory’s account of how microsystem collapse disrupts a child’s capacity for emotional regulation (Bowlby, 1969). When the environments that once scaffolded the child’s development are simultaneously destroyed, the attachment figure, typically the mother, becomes the child’s sole remaining source of safety, placing acute pressure on an already-strained relationship (Bronfenbrenner, 1979).

### Theme 2: parenting after the earthquake

Based on the interviews, parents’ assessments of their parenting skills fall into three categories. While some parents consider themselves competent, most feel inadequate for various reasons (such as impatience, inconsistency, lack of time, and difficulty setting boundaries). This situation demonstrates the negative impact of post-earthquake stress and difficulties on parental self-efficacy. Additionally, a group of parents struggled to assess their competence, remaining undecided by citing both positive and negative examples.

Interviews with mothers reveal that parenting experiences have become significantly more challenging in the post-earthquake period. Many mothers reported not knowing how to respond to their children’s changing behaviors, that their patience was waning, and that they felt inadequate. Behaviors such as crying, stubbornness, tantrums, and disobedience were found to reduce mothers’ tolerance and lead to self-criticism:

*“There’s been a drastic change in my child. She won’t eat at all, and she won’t sit down at the table. She’s constantly outside chasing cats and dogs… Even at school, she’s always crying to her teacher. I’m doing everything I can to get her to go to school. The teacher calls me crying, saying, ‘Come and take her home.’ ‘I don’t want to go to school,’ she tells me… I pick her up from school halfway through the day. She won’t listen at all. She’s so hyperactive… I’m just so exhausted now. It’s really getting to me. My other children weren’t like this. I’m usually such a meticulous, organized mother… This isn’t good at all, and I’m not happy with this situation.” (M34)*

*“We had a more organized life before the earthquake. But right now, since I can’t maintain that order… You can’t keep up, and when you can’t keep up, you eat yourself up inside. Why can’t I do it? Everyone else can maintain order, why can’t I? Everyone else can keep up. Why can’t I? You just eat yourself up inside.” (M13)*

In addition, mothers are experiencing the fear of loss caused by the earthquake and are struggling to cope with it:

*“I started to be a little too overbearing with them, trying to protect them too much, worried that something might happen. Worried I wouldn’t be able to save them. Anxiety, yes” (M10).*

Some mothers have reported struggling to communicate with their children, that what they say no longer has the same effect on them as before, and that they have difficulty establishing authority. In particular, the fact that rules have become unsustainable in container living has made parenting roles even more difficult:

*“The discipline, rules, and order I used to enforce with my children are completely gone–they’ve completely broken down. There’s no trace of our old routine left. The discipline, rules, and household rules I applied to my other two children–I couldn’t apply them to my third daughter, the 5-year-old. I couldn’t instill those effects in her.” (M32)*

Mothers have reported using various disciplinary and coping methods to manage their children. While methods such as communication, modeling, and rewarding are frequently mentioned, some mothers have noted that these methods are insufficient, and that they sometimes resort to harsh reactions, such as deprivation, distraction, changing the environment, and even violence and punishment as a means of coping:

*“Some kids just don’t listen unless you use force. You try to reason with them nicely, over and over again. But eventually, your patience runs out. I don’t usually lay a hand on the kids, but thank goodness for that. They really drive me up the wall sometimes.” (M21)*

*“Usually, when they wash their hands and face and sit down at the table, I say, ‘Good job, you’ve completed this behavior; if you keep it up until tonight, you can have ice cream.’ Hand and face washing and hygiene are important; that’s how I convey it. When he fulfills his school responsibilities and does his homework, I let him play games on my phone or TV for half an hour or an hour. Of course, we choose the games together. I also let him play games I deem appropriate. Aside from that, I buy him whatever he wants as much as I can afford. Right now, my son says, “If I get an A, I want an authentic soccer ball.” So, I’m making and selling dumplings to buy him an authentic soccer ball. In other words, I reinforce their desires and preferences by rewarding them with the things they love, within the limits of my budget.” (M7)*

On the other hand, some mothers said they try to spend as much quality time as possible with their children, noting that playing, reading books, and doing daily activities together benefits both the children and them. However, it appears that these efforts are often limited due to spatial constraints and fatigue:

*“I can’t do much because the space is so small. But we play with small balls. Other than that, we listen to music and dance” (M9)*

“I play with them. Whatever they want, I do. For example, I spread their toys out in front of them. Or I turn on music and play with them. So they don’t get bored. If they say, ‘Let’s go to the store,’ I take them to the store. If they say, ‘Let’s go outside,’ I take them outside. If they say, ‘Let’s go to the park,’ I take them to the park. My day goes like this: I cook, I clean. I take them out.” (M19)

*“To be honest, we don’t spend any time together. Actually, I don’t like this at all; it feels very wrong to me. I can’t find the time. Plus, I don’t even know how to spend time with them. The things I do feel like they’re completely unnecessary to me…” (M15)*

Mothers reported struggling with communication, interaction, and relationships with their children, attributing these difficulties to their own trauma experiences, their children’s changing behaviors, and the constraints of container city living. From a family systems perspective, these difficulties reflect the broader destabilization of the family unit following disaster (Bowen, 1978). As each member’s distress affects the others, the family’s capacity for calm and attuned communication breaks down. In its place, patterns of withdrawal, conflict, and harsh discipline begin to emerge. Mothers who described themselves as previously organized and careful are now parenting under conditions of chronic stress and spatial confinement. This shift reflects not individual failure but a predictable consequence of sustained microsystem disruption (Bronfenbrenner, 1979).

### Theme 3: life in a temporary shelter

Interviews with mothers indicate that life in a container city affects children’s behavior and family relationships. These living conditions entail not only physical constraints but also constant alertness, insecurity, and the need to reorganize daily life.

Many mothers have expressed that they do not consider the container city environment safe enough for their children. Strangers, substance use, traffic and vehicles, violence, and noise prevent parents from letting their children roam freely; this situation restricts the children’s range of movement:

*“I can’t let my children go outside alone. There are all kinds of people here; plus, cars are constantly passing by where I live. Because so many cars go by, I must go out myself. My child can’t go out alone; I’m afraid. There’s even a park, but my children don’t go there often–maybe once a month or every now and then. They can’t go alone either; they go with me. I’m very afraid. All kinds of people are here. This isn’t a safe place.” (M12)*

This insecurity leads to constant interference in mothers’ relationships with their children. The children’s natural play and exploration behaviors are frequently interrupted, and the mothers are forced to adopt a more restrictive attitude, even if they don’t want to:

*“When a guest comes over, there’s no space to play in the container. There are only two rooms anyway. We end up scolding them–whether we want to or not–so our guests aren’t bothered. When the space is cramped, we restrict them: ‘Don’t do this, don’t do that.’ The child is right, but we’re the ones restricting them. If the children had a playroom, for example, we could say, ‘Go on, son, play over there; don’t bother the guests,’ and they’d go there.” (M8)*

One of the most frequently highlighted aspects of container living is the sense of spatial confinement. The lack of private rooms and play areas for children increases family tension and intensifies conflicts, particularly among siblings. Some mothers have stated that due to these spatial limitations, their children have become more irritable, impatient, and intolerant, creating a constant tension within the home:

*“It’s not like being at home here; we’ve really struggled because there are six of us… My kids just won’t adjust to this place–they won’t even throw out their trash anymore; they just don’t want to. At home, for example, they used to clean their rooms; now they don’t even do that. I feel inadequate in that regard. Because whenever something happens, even my daughter says, ‘Mom, do I have a room? Should I clean it?’ or when it’s time to study: ‘Do I have a desk, Mom?’ So they’re always asking me about a home environment…” (M5)*

*“It’s impossible for five people to live here… They put you in a tiny room and tell you to live there, five people. For example, if you get fed up, you have no chance to move to another room. And you’re still forced to stay in the same space with them. You can’t even storm off and leave. So when you argue with your spouse, you can’t go to another room. You’re forced to stay in the same space and keep going. And that just makes the situation worse.” (M29)*

*“In such a cramped space, there’s more fighting and noise… They have disagreements with each other. A friend of mine who’s raising twins says, ‘I didn’t have it this hard.’ They’re more aggressive than twins.” (M21)*

It has also been frequently noted that life in the container city disrupts family routines, makes daily life more difficult, and increases the burden on parents. Economic hardships, instability, and increased responsibilities heighten tension within the family, which indirectly affects the children’s behavior:

“We’re in a small space; inevitably, our sleep schedule, going to bed, waking up–everything has changed here…. Housing problem. We have a food problem. For example, when we were at home, we cooked every meal, but here it’s very hard to use the space for both sitting and sleeping. I tell the kids to go to bed, but they can’t. It’s often 1 or 2 a.m. before they fall asleep. Because there’s limited space and it’s cramped.” (M18)

*“There are no study spaces for the children… For instance, my spouse and I can’t have a private conversation about anything. The children have no place to study. Sometimes, for example, when one wakes up, the other wakes up too–everyone in the house wakes up from the noise. When there are issues with electricity or water in the container, we get very stressed.” (M35)*

In this context, the container city is not merely a shelter; it emerges as a powerful environmental factor shaping children’s and parents’ behaviors, as well as family relationships. In this sense, the container city functions as a chronic environmental stressor that fundamentally transforms parenting, turning it from a relationship-based practice into a constant effort to manage risks and restrictions.

### Theme 4: needs

The needs mothers expressed in this study reflect four overlapping pressures: the unsafe and unstable conditions of container city life, mothers’ own unaddressed trauma and exhaustion, children’s ongoing emotional and behavioral difficulties, and the lack of adequate institutional support. These pressures feed into each other and cannot be addressed separately. The needs identified in this theme fall into five areas: emotional regulation, safety, relational repair, developmental knowledge, and institutional support. Interviews with mothers reveal that the difficulties children face in the post-earthquake period are not viewed merely as individual or temporary problems but rather as a need for multifaceted, ongoing support for both children and mothers. The needs expressed under this theme have been categorized into personal support and parenting quality support.

The constant state of vigilance, economic anxieties, and children’s increasing needs are depleting parents’ emotional resources:

*“While struggling with so many things in the areas where we’re struggling and trying to be perfect, I’m actually hiding my own wounds in the background. At some point, those wounds inevitably cause me tension and stress. When it comes to mind, I consult our teachers here to avoid conflict. I seek support, and in areas where I can’t get support, I usually try to resolve things by talking with the children” (M7)*

*“Frankly, I’d like to receive education regarding myself and my children. Because if I improve, they’ll improve anyway. You know how they say, ‘If the woman is happy, the family is happy’? It’s exactly like that.” (M9)*

Mothers have reported that, in addition to developing emotional regulation and flexibility skills for their own well-being, they need to build positive relationships with their partners and access social support and social activities.

*“I’m very meticulous, very obsessive. Or maybe I’m just very organized, very strict. I say yes when I should, and no when I should. I know exactly where and when to say yes or no. But sometimes, when I say no, I stop and ask myself: Am I being too strict? Am I going too far? Should I be more flexible?” (M22)*

*“I’ve always had to struggle on my own. Unfortunately,” I handle the household needs, the children’s needs, and their emotional needs all by myself. (M25)*

*“I yell a lot. I get angry quickly. I’m strict. My older daughter says, ‘Mom, you’re so good, but you yell a lot.’ Things don’t always go the way we want them to, but that’s just how it is. …” (M34)*

The interviews with mothers also revealed a need for parenting support to meet children’s needs. Some mothers stated that they realized the emotional difficulties their children were experiencing were not something they could handle on their own and that they needed professional support. This search for support is seen as a source of relief for both the child and the parent:

*“They’ve become more hyperactive, more irritable, aggressive–they’ve started acting out. That’s why I make sure they participate in activities. We sought support from psychologists and met with them. We’ve started to recover, and we’ll continue to do so.” (M7)*

In the mothers’ accounts, the children’s needs regarding socialization and peer relationships also stand out clearly. It was noted that children who cannot form friendships in a safe environment often exhibit withdrawal or crying. The mothers stated that they did not know how to socialize their children or how to support this process:

*“He wants a friend but can’t make one. For example, a child who plays alone when we go outside. He likes to play by himself. But sometimes he sees a friend. For example, he says: ‘Mom, this one won’t play with me.’ He sits there and cries.” (M20)*

Many mothers have reported struggling to help their children regulate their emotions and feel unsure about how to approach the situation. In particular, persistent crying, anger, and fear leave mothers feeling helpless, and they often feel uncertain about what constitutes appropriate parenting:

*“How should I approach my child? For example, it would be good if my son came over a little. I mean, how can I help him socialize? I want him to play like this. I want him to talk and express himself, for instance. How can I encourage positive behavior? I want him to stop crying so much.” (M18)*

Some mothers have noted that issues such as low self-confidence, difficulties with sharing, and sibling jealousy have become more apparent in their children following the earthquake. It was mentioned that these situations are linked to both spatial constraints and a state of constant stress:

*“They’ve started becoming extremely aggressive and selfish. They’ve stopped sharing much. Fights have increased.” (M21)*

*“They also can’t defend themselves. You know, when older kids from their own age group hit them, they immediately feel the need to complain. There’s a bit of a lack of self-confidence. I thought that as their social circle expanded here, their self-confidence would grow. But my older son is the one who complains more about this.” (M13)*

The interviews also discussed the challenges mothers face in their relationships with their children and their need for educational support. A significant portion of the mothers emphasized their need for information and guidance regarding healthy communication with their children and appropriate parental attitudes and behaviors.

*“I’d like to learn how to communicate with my children–you know, they don’t listen to me–how can I get them to listen more?” (M2)*

*“I don’t know how to approach my child or how to behave around them.” (M25)*

Additionally, it is evident that mothers lack knowledge about child development and experience uncertainty regarding which behaviors are age-appropriate and which are related to trauma:

*“Their language is crude and vulgar. For example, they attack their food. I want my child to improve… they’re not an ignorant child… But why are they acting this way? I just can’t wrap my head around it.” (M34)*

*“How does one become a more patient mother? I mean, what does it really mean to play effectively with a child, to spend quality time with them? I’m really curious about this.” (M33)*

In addition to these, they expressed their need to support their children by sharing the difficulties and concerns they face regarding child psychology, behavioral issues, emotional regulation, child health and nutrition, technology use, substance use, and privacy:

*“It could be about development or nutrition, or it could be about conversations that might affect the child psychologically.” (M4)*

*“My little son is scared. I tell him, ‘Son, there’s nothing to be afraid of; we’re in a tiny space, and we’re all together. Why are you scared?’ He’s afraid of the elevator or something like that. How can I comfort him? Sometimes I lie down next to him, and sometimes I tell him, ‘You’re a big boy now,’ because I want him to feel more at ease when we’re in a home environment.” (M5). It could be education about how a child behaves toward the mother and how the mother behaves toward the child. Then there’s violence. Because domestic violence happens a lot in the container… Then there’s addiction. It could be about safety. It could be about not trusting others. You know, when we let our kids play in the park… they lure them away by saying, “Come here, I’ll give you candy” (M20)*

*“When I hear the word ‘education,’ the first things that come to mind are sexual abuse, substance abuse, and privacy. The most important of these is how we explain these things to our children. Whatever technology is, as a mother, I might be able to explain that somehow. But how, for example, will I explain privacy to my child? … The child is also imagining this while you’re explaining it, after all.” (M32)*

Many mothers believe they cannot support their children’s education as they did before the earthquake, and due to both financial difficulties and the lack of a suitable environment, they feel their children are falling behind:

*“My children had their own rooms and living spaces, but suddenly they lost everything. For the first week, we struggled to find food, bread, and water; we faced many financial hardships. While I was arranging private lessons for their education, I could no longer provide them with the special support they needed; I could barely meet their basic needs. We’re still struggling.” (M7)*

*“They can’t relax because of the stress of doing homework. Their grades have slipped, for example. It used to be different… their communication has changed. The outside world has a big impact.” (M9)*

Some mothers have noted that they’ve realized structured activities are very beneficial for children, but they don’t know how to plan or sustain such activities. It appears that play, reading books, and some small activities help soothe the children, but parents need guidance in this regard:

*“I’d like to get them something nice. I even thought about getting a big chalkboard. I wanted to teach them the alphabet before they started preschool. I had them memorize some nice words and poems. I wanted them to draw. So they’d do this instead of misbehaving.” (M21)*

Mothers indicated that both their own needs and their children’s needs had become highly diverse, with parenting skill development emerging as a prominent area of support. The needs identified in this theme reflect disruptions at all three theoretical levels. At the ecological level, the loss of a stable living environment has removed the conditions that once supported everyday parenting. At the attachment level, mothers’ own unaddressed trauma has reduced their capacity to provide emotional security for their children. At the family systems level, accumulated stress has damaged the communication patterns that once held family life together. Meeting these needs, therefore, requires support that addresses all three levels at once.

## Discussion

This study examined the effects of the February 6 earthquakes on the lives of mothers who experienced the Kahramanmaraş earthquake in Türkiye in 2023 and were living in a container city, with a particular focus on their parenting experiences and needs to rebuild their lives.

### The impact of the earthquake on children

The findings reveal that earthquakes have multifaceted and long-term effects on children, that these effects are even more pronounced in container cities, and that mothers need psychological well-being support for both themselves and their children. Furthermore, the study indicates that post-earthquake recovery should be understood not merely as an individual psychological process, but as a relational and socio-ecological process shaped by care and living conditions, as well as the continuity of the social and supportive environment. The study emphasized the importance of considering the simultaneous emergence of psychosocial developmental disorders in both children and mothers.

The study highlighted the importance of considering the co-occurrence of psychosocial developmental disorders in children and mothers. In the study, mothers reported that their children had experienced negative emotional and behavioral changes following the earthquake. These included anxiety, fear, shyness, and outward behaviors such as outbursts of anger, aggression, irritability, and defiance. The earthquake’s effects continued to affect children both during and after the event. In this context, Eroğlu and Yaksı (2025) found that children and adolescents exhibited behavioral difficulties following the earthquake in their long-term follow-up study. Although recovery from a disaster is expected to take time, being separated from one’s previous living environment can also prolong it. Kurt et al. (2024) have highlighted that children of parents affected by the earthquake exhibit significant behavioral issues and emotional distress, with the condition of their homes being a key contributing factor. Özyurt et al. (2025) focused on psychiatric characteristics in earthquake-affected children and emphasized that externalizing behaviors are among the most common post-traumatic responses. These behavioral changes are thought to reflect children’s difficulty in regulating their emotions after trauma (Pine and Cohen, 2002). While individual responses to traumatic events vary from child to child, the consistency of the fundamental response is clear: increased anxiety, general fears, and loss of self-esteem (Garbarino, 1992). Children express the intense fear, danger, and insecurity they experience through immature emotional regulation skills, manifesting as aggression, outbursts of anger, and oppositional behavior. The behavioral problems exhibited by children following an earthquake may also stem from the psychological difficulties their mothers experienced after the earthquake. Indeed, Fukuchi et al. (2026) found that parents’ subjective perceptions of earthquake-related damage were significantly associated with their own psychological distress, which in turn was linked to emotional and behavioral problems in children born after the earthquake.

In the study, mothers reported that children’s habits-such as sleep and restroom use-were lost or deteriorated following the earthquake. Disruptions in children’s routines–such as their sleep and toilet habits–can be seen as a sign that the earthquake has also affected them developmentally. A regression in a skill a child had previously acquired can reflect a reaction that arises when the child feels unsafe and struggles to cope with stress. Similarly, in a study by Koçak et al. (2025), most children reported sleep disorders following the Kahramanmaraş earthquake. Another study reported that children exhibited behaviors such as fear, sadness, stress, loss, displacement, sleep disturbances, persistent recollection of the earthquake, and avoidance of school following the earthquake (Özer et al., 2024).

In container cities, a “home” is not merely a place of shelter for children, but also a living space where they find security, a sense of belonging, and the continuity of daily routines. Therefore, the transition to a permanent and safe environment can support children’s psychosocial well-being. Disasters disrupt the socio-spatial bonds of the people and communities they affect (Berroeta et al., 2024). In a study by Şen and Şen (2026), it was found that following the twin earthquakes centered in Kahramanmaraş on February 6, 2023, the meaning that young people living in Malatya ascribed to the concept of “home” underwent a profound process of restructuring at the spatial, emotional, and social levels. Before the disaster, “home” was defined by comfort, privacy, security, memories, and family; after the disaster, it was found to have transformed into a place where trauma, uncertainty, and a perception of chronic risk were experienced, and it was viewed more as a temporary shelter than as an environment for daily life. Scannell and Gifford’s (2010) study revealed that homes are superior to other types of housing in providing individuals with physical and psychological comfort and a sense of security. In this context, moving children from container cities to permanent housing may change the meaning they ascribe to “home” and support their psychological well-being.

In the study, mothers expressed their children’s longing, searching, and questioning regarding the loved ones they had lost. The children’s questions, such as “Where is he now?” and “Will I see him again?,” indicate that they struggled to make sense of the sudden loss and exhibited grief reactions. Children’s repeated questions about people they have lost may reflect not only their grief but also an attempt to make sense of the event, reduce uncertainty, and regain a sense of safety. For this reason, how adults respond to children’s questions is particularly important in helping them develop a sense of security following a traumatic event. Because young children are still developing an understanding of death and its permanence, they may have difficulty fully comprehending what has happened and may repeatedly ask the same questions as they try to process the loss. Clear, honest, and developmentally appropriate responses can therefore help children make sense of the loss while providing reassurance and emotional security (Coates and Schechter, 2004). As Cohen et al. (2006) noted, children who have experienced trauma may become fixated on the traumatic aspects of a loved one’s death, and as they begin to recall their loved ones, even happy memories can transform into thoughts about the person dying in a horrific and terrifying manner. This situation may also lead them to avoid remembering their loved ones (Cohen and Mannarino, 2011). Bozkurt Karalı and Özkan (2025) have highlighted that children who have experienced a recent loss exhibit more intense post-traumatic stress symptoms and that the mother-child relationship plays a significant role in this process. Instead of avoiding conversations about the loss, mothers should provide developmentally appropriate, open, and consistent explanations; express the irreversibility of death in concrete and simple terms; and allow space for the child’s emotional expressions–all of which are critical in reducing the intensity of traumatic grief. This approach supports long-term psychosocial adjustment by helping children cognitively process loss and maintain emotional regulation skills within a secure attachment relationship (Assadi, 2023; Wray et al., 2026).

### Post-earthquake parenting experiences

The study also provided findings on the earthquake’s impact on the role of motherhood. In a study based on professionals’ observations of post-earthquake life, mother-child communication was emphasized, and mothers’ child-rearing roles were highlighted (Temşi, 2024). In disaster situations, women typically take on domestic responsibilities, while men assume financial responsibilities (Villarreal and Meyer, 2019). Mothers’ isolation in managing household responsibilities and childcare, coupled with suppressing their emotions during this process, can negatively affect their emotional regulation skills and reduce parenting quality (Crandall et al., 2016; Töz et al., 2023).

When comparing parent-child interactions to those before the earthquake, mothers emphasized that they experienced a significant decline in their parenting self-efficacy due to trauma, felt unsure of what to do in their relationships with their children, and felt inadequate. Coleman and Karraker (2003) state that low parenting self-efficacy is associated with increased anxiety. Kerimoğlu Yıldız et al. (2024) found that 1 year after the Kahramanmaraş earthquake, parents continued to experience anxiety and depression, and that this situation also influenced the emergence of psychosocial problems in their children. Kuru and Ungar (2025) found that parents’ psychological difficulties after the earthquake were positively associated with their children’s psychological difficulties and negatively associated with their children’s prosocial behaviors. Uçar Çabuk (2023) found a negative relationship between mothers’ earthquake anxiety and their well-being; it was observed that mothers who were trapped under rubble or experienced the loss of relatives had high levels of earthquake anxiety and low levels of well-being.

Mothers reported experiencing intense fear of loss following the earthquake, which manifested as overprotective behavior. Earthquake trauma continues to trigger prolonged anxiety and fear because it separates individuals from their safe spaces, and the ongoing shaking ensures that the sense of danger persists. In this context, securing permanent housing and transforming living environments into spaces that foster a sense of safety are crucial.

It has been determined that, in addition to positive disciplinary methods such as communication and modeling, mothers also use negative methods such as deprivation and punishment toward their children after an earthquake. Furthermore, factors such as lack of time and space, as well as physical and mental fatigue, have been identified as barriers to spending quality time with their children.

These negative behaviors on the part of mothers necessitate an assessment of post-disaster parenting processes within the context of trauma and chronic stress. Intense stress, economic uncertainty, and loss of social support can complicate parents’ emotional regulation processes and increase the risk of harsh parenting (McDermott and Cobham, 2012). However, this does not imply that violence against children is normalized. Child protection risks may increase during post-disaster periods; therefore, child protection mechanisms must be strengthened through trauma-informed psychosocial support (UNICEF, 2024). In this context, it is crucial that family support programs be structured with a comprehensive approach that prioritizes child safety while providing supportive services without stigmatizing parents.

Yumbul et al. (2018) found that mothers’ coping skills deteriorated after the earthquake; they struggled to manage their negative emotions and became more punitive. This change led to situations such as children’s disobedience and imitation of the mother’s negative behavior. On the other hand, they noted that counseling services for mothers helped them form emotional bonds with their children, regulate negative emotions, and develop effective parenting skills. In another study conducted with families affected by the earthquake, parents reported using physical punishment, and this was associated with children’s internalizing, externalizing, and behavioral problems (Miki et al., 2019). It can be said that parents’ struggle with their own traumas also affects their relationships with their children and leads them to adopt authoritarian attitudes toward their children. The regular and continuous provision of psychological support services following a disaster will enable mothers to cope with their own anxieties and facilitate their ability to fulfill their parenting roles more effectively.

### Life in the container city: constricted setting, less security and less privacy

Study results revealed that mothers described living in container cities as involving not only physical limitations, such as restricted living space and a lack of privacy, but also ongoing feelings of insecurity, uncertainty, and emotional strain. Although the shelters were located within a gated community with controlled access, the mothers did not perceive this environment as safe. Previous studies have also highlighted security concerns in temporary housing and the lack of dedicated spaces for children (UNICEF, 2024). The mothers explained this as the random placement of families within the facility, not knowing one another, and significant cultural and language differences. They noted that security concerns prevented them from letting their children play freely, and that the container home environment, limited to 21 square meters, did not meet the children’s need for play.

A striking finding of the study is that the mothers used terms such as “here” or “container” instead of “home” to describe their temporary living situation, indicating that they did not feel a sense of belonging. Furthermore, during the interviews, the mothers expressed their attachment to and longing for their destroyed homes by using phrases such as “when I was at home” or “when I was in my home.” These statements may also be interpreted through the concept of belonging. A sense of belonging is closely associated with feelings of safety, stability, connectedness, and emotional security within one’s social and physical environment. For children and families living in container cities, the loss of home and familiar social environments may weaken feelings of attachment and a sense of normalcy. Additionally, disrupted social support networks, feelings of insecurity, and the loss of daily routines can significantly affect family functioning and parent-child relationships after a disaster (Fothergill and Peek, 2015). Mothers’ difficulties in sustaining family order and supporting children’s emotional needs may therefore reflect not only practical caregiving challenges but also efforts to reconstruct a sense of belonging and emotional security under temporary and uncertain living conditions.

Traumatic experiences are shaped not only by the event itself but also by the physical and social environments in which they occur (Evans, 2006). This perspective is consistent with Bronfenbrenner’s (1979) Ecological Systems Theory, which emphasizes that children’s development and family well-being are embedded within and influenced by their immediate physical and social environments. From this perspective, the social quality of the post-disaster environment may play an important role in families’ recovery. Borinca et al. (2025a) highlight that inclusion within a valued overarching group can reduce discrimination, dehumanization, and unjust targeting. Therefore, post-disaster recovery should be considered not only in terms of parenting capacities or trauma-related symptoms but also in terms of whether temporary living environments provide families with a sense of social inclusion, dignity, and responsiveness to their needs. The feeling of being part of a community, being recognized, valued, and supported, can accelerate mothers’ well-being. In this context, maintaining routines, preserving family relationships, and creating emotionally supportive interactions become important mechanisms through which mothers attempt to restore stability and continuity in their everyday lives. Providing safe, child-friendly environments for families will benefit both families and children by reducing post-disaster stress and fostering a sense of belonging. The post-disaster recovery process is not unidirectional or linear; rather, individuals’ economic conditions, social relationships, housing security, and sources of social support play a decisive role in psychological recovery (Bonanno et al., 2010; Hobfoll et al., 2007). Accordingly, post-disaster policies should prioritize the rapid transition of earthquake-affected families from temporary shelter to safe, sustainable permanent housing.

### Well-being, parenting and children’s needs

In the mothers’ statements and examples regarding the earthquake’s impact on them, they emphasized that they were still affected by the earthquake and required emotional and social support. Studies on post-disaster family life indicate that prolonged displacement, temporary housing conditions, and chronic environmental stress can negatively impact parents’ psychological well-being and caregiving processes (Masten and Narayan, 2012; Prime et al., 2020). During the first year following the earthquake, psychologists, psychological counselors, and social workers provided emotional and social support to earthquake survivors in temporary shelters. However, the magnitude of the trauma and not yet having returned to their homes underscore the need for ongoing emotional and social support.

Mothers stated that despite having experienced trauma themselves, they are aware of traumatic changes in their children and make efforts to comfort them. They are questioning their parenting and their children’s educational needs. Their children’s educational needs included emotional regulation, self-regulation, a safe environment, positive social relationships, self-care, self-esteem, communication, effective parenting attitudes and behaviors, child psychology, health and development, attachment, safety, privacy, special needs, and structured activities. These needs encompass contexts aimed at regulating the mother-child relationship. The needs mothers highlighted for themselves and their children also reflect the need for a comprehensive welfare intervention program.

Bowen’s Family Systems Theory posits that family members operate within an emotionally interconnected system and that chronic stress conditions can affect family functioning, potentially influencing parental sensitivity, boundaries, and emotional regulation processes (Bowen, 1978). From this perspective, the constant environmental pressures of temporary housing conditions do not merely generate individual stress; they also shape mothers’ perceptions of their parenting competence and children’s behavioral adjustment. The mothers participating in this study were relocated to a container city immediately after the earthquake and have been trying to continue their lives there for approximately 22 months.

The findings can be understood through Bronfenbrenner’s ecological perspective, which emphasizes that children’s development and family well-being are shaped by interconnected environmental systems rather than by individual or family experiences alone (Bronfenbrenner, 1979; Bronfenbrenner and Morris, 2006). The present study indicated that the fact that mothers had been away from home for approximately 2 years suggests that the effects of the February 6 earthquakes had extended beyond acute trauma responses and become embedded in the ongoing developmental trajectories of mothers and children. Changes in the family’s living environment may have disrupted routines, altered parenting attitudes, and increased caregiving burdens, and the resulting difficulties may reflect both earthquake-related trauma and ongoing stress from changes in living conditions (Cui, 2013; Triana, 2018). These experiences can be considered across ecological levels: disruptions in the microsystem may affect children’s sense of safety and family interactions, while exosystem conditions, including changes in parents’ work and access to community and social services, may indirectly influence family functioning. At the macrosystem level, broader social, economic, cultural, and institutional conditions may shape families’ access to resources and opportunities for recovery, whereas the chronosystem highlights the cumulative effects of a major historical event such as the February 6 earthquakes over time (Bronfenbrenner, 1979; Bronfenbrenner and Morris, 2006; Fiala et al., 2025). Within this ecological context, psychosocial interventions are important for supporting both mothers and children. Evidence indicates that trauma, anxiety, and other psychological difficulties may persist following earthquakes (Chen et al., 2020; Cui, 2013; Dorahy et al., 2016; Tanaka et al., 2016; Tang et al., 2020), while parental psychological difficulties may also be associated with continued psychosocial problems among children (Chen et al., 2014; Wang et al., 2000). Therefore, interventions should not focus exclusively on individual symptoms but should also strengthen the relational and social environments surrounding families. Given evidence that children need support following traumatic events (Özer et al., 2024), social support (Kaya et al., 2025) and meta humanization-based interventions (Borinca et al., 2025b) may contribute to recovery. From an ecological perspective, supporting mothers may extend beyond individual well-being by strengthening the microsystem in which children develop and by enhancing families’ capacity to navigate broader systems during long-term recovery.

## Conclusion and recommendations

This study identified the experiences and needs of mothers living in a container city following an earthquake. The findings indicate that the difficulties mothers experience in their parenting processes cannot be explained solely by individual parenting skills. Lack of living space and privacy, economic uncertainty, security concerns, and limited psychosocial services are significant structural factors that complicate mothers’ caregiving. These findings are consistent with Bronfenbrenner’s Ecological Systems Theory, which emphasizes that children and families are embedded within interconnected environmental systems and that changes in these systems can influence family functioning (Bronfenbrenner, 1979).

The fact that the data for this study were collected long after the earthquake, during a period when life in the container city was still ongoing, is significant for interpreting the findings. The findings reflect not only the effects of acute earthquake trauma but also the chronic psychosocial burden resulting from prolonged displacement, temporary housing conditions, and ongoing environmental uncertainty. From an ecological perspective, the continued disruption of the family’s immediate environment may influence parenting, family relationships, and children’s adjustment over time. Thus, the findings extend the focus from the earthquake’s immediate consequences to the ongoing effects of environmental disruption on family life. In this context, our main recommendation is the urgent placement of families in permanent housing to foster a sense of belonging and mitigate the effects of trauma. This would also resolve issues such as privacy and security arising from mothers living in container cities. Furthermore, developing and implementing intervention programs to support both the well-being and parenting attitudes of earthquake survivors until they are settled in permanent housing would also be beneficial from a public health perspective. In light of the concerns identified in the study regarding physical conditions, guidelines and regulations for planning and implementing temporary housing can be developed to prevent trauma during the processes of relocating disaster survivors and providing support services.

This study highlighted that mothers’ needs after the earthquake fell into two categories: personal well-being and parenting needs. Their well-being needs included emotional regulation, social support, a healthy relationship with a spouse, social activities, a safe environment, and friendship. Parenting needs included healthy communication with the child, parental attitudes and behaviors, child health and nutrition, child protection/safety, and privacy. These findings can also be understood through attachment theory, which emphasizes the importance of caregivers as sources of safety and emotional regulation, particularly when children experience fear, uncertainty, and threat (Bowlby, 1969). It is difficult to distinguish these needs from one another and prioritize them by importance. For example, a mother whose need for social support is met will also experience positive changes in her family relationships. This interconnectedness is consistent with Bowen’s Family Systems Theory, which conceptualizes family members as interdependent and suggests that changes in one family member can influence the functioning and well-being of other family members (Bowen, 1978). In this study, mothers’ personal well-being and parenting needs were similarly interconnected rather than separate domains. A mother’s access to social support, emotional well-being, and relationship with her spouse may influence her ability to respond to her child’s needs, while difficulties in parenting and concerns about the child may, in turn, affect the mother’s own well-being. Thus, the findings support the view that post-disaster maternal experiences should be understood within the broader family system rather than as individual experiences.

The findings also highlight the gendered dimensions of post-disaster recovery. Mothers were expected to maintain caregiving responsibilities, regulate children’s emotions, and preserve family functioning despite their own uncertainty and emotional distress. This reflects broader sociocultural expectations surrounding motherhood, particularly within contexts where women are positioned as primary caregivers responsible for emotional and familial stability. Accordingly, motherhood in post-disaster settings should be understood not only as an individual parenting experience but also as a socially structured and gendered process shaped by cultural expectations and structural conditions.

Taken together, the findings support an integrated interpretation of post-earthquake family experiences. Ecological Systems Theory helps explain how prolonged disruption of housing and community environments contributes to family stress; attachment theory highlights the importance of mothers’ capacity to provide safety and emotional regulation for children; and Family Systems Theory explains how maternal well-being, parenting, family relationships, and children’s adjustment are interconnected. Considering these perspectives together suggests that interventions targeting mothers and children should not focus exclusively on individual psychological adjustment or parenting skills. Instead, effective post-disaster support should address the relational, family, and environmental conditions that shape mothers’ and children’s recovery.

For future studies, it may be recommended to examine the experiences of mothers living in different provinces affected by the earthquake and under various temporary housing conditions. Longitudinal studies could allow a more detailed examination of the long-term effects of earthquake trauma on parenting attitudes, child development, and psychosocial well-being. Using multiple data collection techniques, such as observation, field notes, and mixed methods design, could broaden data diversity. Including the perspectives of fathers, other caregivers, and children could contribute to a more comprehensive assessment of the family system. To better assess the effects of earthquakes on children, studies could include direct interviews with children, use natural observation data, and incorporate reports from teachers or caregivers. Interdisciplinary, planned, and systematic studies must be conducted to fulfill social and emotional needs to mitigate the long-term effects of earthquake trauma on the children and parents.

### Limitations

The February 6 earthquakes are among the largest to cause destruction in 11 provinces in Türkiye. Because of transportation and financial limitations, researchers interviewed only mothers living in a container city in Osmaniye. Consequently, it would not be appropriate to generalize about conditions and experiences in other provinces.

Mothers who volunteered to participate in the study may be emotionally prepared to share their experiences. Mothers who have suffered significant loss of life or property and are experiencing related trauma may be reluctant to participate in the study.

A potential limitation of this study is the possibility of self-selection bias. Mothers who volunteered to participate may have been more emotionally prepared or willing to share their experiences than mothers who chose not to participate. In particular, mothers who experienced substantial loss of life or property and who continued to experience significant trauma-related distress may have been less likely to participate in the study or discuss their experiences. Therefore, the findings may not fully represent the experiences of mothers who were experiencing the most severe levels of post-earthquake distress.

Additionally, the study is limited to mothers with children aged 4–6. The findings provide data on children and mothers in early childhood. Because the study included only mothers of children aged 4–6 living in a container city, the findings’ generalizability to different disaster contexts and sociodemographic groups is limited. The reliance on mothers’ self-reports, without including the perspectives of fathers or other caregivers, limits the study’s ability to assess family-system experiences multidimensionally. The study’s failure to incorporate observation, include different participant groups, or utilize longitudinal data collection methods–all of which could have increased data diversity–also represents a limitation. Both the age group and the nature of the qualitative findings limit generalizability. Future studies could enhance generalizability by including different age groups and using alternative research methods.

## Data Availability

The data obtained in this study were collected within the scope of a project, subject to confidentiality principles. The data may be obtained in part from the corresponding author upon reasonable request.
